# Species Richness Responses to Structural or Compositional Habitat Diversity between and within Grassland Patches: A Multi-Taxon Approach

**DOI:** 10.1371/journal.pone.0149662

**Published:** 2016-02-22

**Authors:** Szabolcs Lengyel, Eszter Déri, Tibor Magura

**Affiliations:** 1 Department of Tisza Research, Danube Research Institute, Centre for Ecological Research, Hungarian Academy of Sciences, Bem tér 18/c, H-4026 Debrecen, Hungary; 2 Zoological Society of London, Regent's Park, London NW14RY, United Kingdom; 3 Department of Ecology, University of Debrecen, P.O. Box 400, H-4002 Debrecen, Hungary; National University of Mongolia, MONGOLIA

## Abstract

Habitat diversity (spatial heterogeneity within and between habitat patches in a landscape, HD) is often invoked as a driver of species diversity at small spatial scales. However, the effect of HD on species richness (SR) of multiple taxa is not well understood. We quantified HD and SR in a wet-dry gradient of open grassland habitats in Hortobágy National Park (E-Hungary) and tested the effect of compositional and structural factors of HD on SR of flowering plants, orthopterans, true bugs, spiders, ground beetles and birds. Our dataset on 434 grassland species (170 plants, 264 animals) showed that the wet-dry gradient (compositional HD at the between-patch scale) was primarily related to SR in orthopterans, ground-dwelling arthropods, and all animals combined. The patchiness, or plant association richness, of the vegetation (compositional HD at the within-patch scale) was related to SR of vegetation-dwelling arthropods, whereas vegetation height (structural HD at the within-patch scale) was related to SR of ground-dwelling arthropods and birds. Patch area was related to SR only in birds, whereas management (grazing, mowing, none) was related to SR of plants and true bugs. All relationships between HD and SR were positive, indicating increasing SR with increasing HD. However, total SR was not related to HD because different taxa showed similar positive responses to different HD variables. Our findings, therefore, show that even though HD positively influences SR in a wide range of grassland taxa, each taxon responds to different compositional or structural measures of HD, resulting in the lack of a consistent relationship between HD and SR when taxon responses are pooled. The idiosyncratic responses shown here exemplify the difficulties in detecting general HD-SR relationships over multiple taxa. Our results also suggest that management and restoration aimed specifically to sustain or increase the diversity of habitats are required to conserve biodiversity in complex landscapes.

## Introduction

Understanding the factors influencing species diversity is central in ecology and biodiversity conservation. Species diversity at global or continental scales is primarily influenced by temperature/solar radiation/energy and water [1]. At regional scales, area effects (species-area relationship, SAR) become more important, and at progressively smaller scales, the importance of habitat diversity (spatial heterogeneity within and between habitat patches within a landscape, HD) relative to area increases [2, 3]. At local scales, HD is primarily important in explaining patterns in species diversity [4]. The SAR suggests that SR increases with area because more species will inhabit larger areas. However, larger areas usually also contain more habitats, provide more niches and can hold more species [5]. Thus, the effect of area cannot be easily separated from HD, and the two effects should be viewed as complementary [6].

In contrast with SAR, the role of HD in explaining patterns in species diversity is much less understood. First, the HD hypothesis lacks a general framework and a central descriptive model [7, 8]. Second, there is confusion over the terms used to describe HD. For example, a review of 85 studies of animals from 1963–2003 [9] found 13 terms and a wide range of variables describing HD. Studies conducted on oceanic islands and archipelagoes often used elevation as a proxy for HD [10, 11], whereas studies of terrestrial habitat islands used some typology of habitats [5], vegetation [12, 13] or soil [14]. Habitat structure, often measured as the standard deviation of vegetation variables, has been incorporated in more recent studies, many of which were conducted in forests [15–18]. Another problem is that different taxonomic or functional groups can show different SR-HD correlations. For example, SR in different families of forest beetles can show increases, decreases or no changes with the structural complexity of habitats [17]. Moreover, some taxa may be influenced by compositional factors of HD (e.g. basal rock types or vegetation types), whereas others may be influenced by structural factors of HD (e.g. configurational complexity, three-dimensional architecture), which factors are typically not addressed separately. Some taxa may be affected by small-scale variation within habitat patches, whereas others may be affected only by larger-scale variation among habitat patches in a landscape [9]. Finally, many studies measured species diversity of one taxon only [19] or for a few taxa separately [6] and we know little on how relations between SR and HD scale up when data from several taxa are combined. As a result, the conclusions of previous studies are split regarding whether species diversity is influenced by HD [20–22], by area [15, 18, 23], or by both effects independently [5, 14, 19, 24] or in interaction with one another [25–27].

Here we test the effects of compositional and structural factors of HD on SR in multiple taxonomic groups in a wet-dry grassland habitat complex. We defined habitat diversity in a wide sense as the spatial heterogeneity within and between habitat patches in a landscape, which can arise from differences in the composition, structure or function of the habitat types present [28]. We first develop a conceptual framework, which incorporates compositional and structural factors of HD at two scales, within and between habitat patches. We then demonstrate how different factors of HD influence SR of taxonomic groups individually, in various combinations of groups or in all groups combined. We specifically asked the following question: is SR influenced primarily by configurational, compositional or structural characteristics within and among the habitat patches? We sampled six taxonomic groups (flowering plants, grasshoppers-katydids Orthoptera, true bugs Heteroptera, spiders Araneae, ground beetles Carabidae, and birds Aves) selected to represent major trophic (herbivores, predators) and taxonomic groups (from plants to birds). We chose several arthropod taxa and only one vertebrate taxon as the former are usually underrepresented in studies of diversity patterns [9], even though they comprise more than 81% of the animal species described thus far [29].

We specifically tested SR-HD relationships by developing a set of alternative general linear models to explain SR by compositional and structural factors of HD at two scales, between and within habitat patches, and then compared the support for these models using information theoretic criteria. Based on the fundamental determination of plants by abiotic factors [5], we hypothesised that plant SR will be influenced by configurational characteristics of the patches (e.g. patch area, shape, isolation, see definitions in Methods). Based on previous animal studies [9, 30], we hypothesised that the SR of vegetation-dwelling, mainly herbivorous arthropods will be more affected by compositional factors of habitat diversity, whereas that of ground-dwelling, mainly predatory arthropods will be more influenced by the structural factors of habitat diversity. For the feeding of herbivores, the floristic composition of plants, i.e., a wider resource base, was expected as primarily important [31–33], whereas for predators, the complexity of vegetation providing microhabitats suitable for predation was expected as primarily important [34]. We tested these hypotheses using an extensive dataset from taxon-specific surveys of multiple taxa in a marsh-grassland ecosystem with traditional low-intensity management by mowing and cattle-grazing.

## Materials and Methods

### Definitions: a framework for grassland habitat diversity

In a wide sense, we define habitat diversity as the spatial heterogeneity within and between habitat patches delineated within a landscape. Differences among habitats arise from the different composition, structure, or functioning of the habitat types present in the landscape [28, 35]. In this study, we focus on compositional and structural habitat diversity (Table 1). Composition refers to the pattern that landscapes consist of habitats of different type or identity (e.g. grassland, wood pasture, forest etc. [28]). We thus interpreted “composition” between patches as the list of different wet and dry habitat types present in the landscape. In grasslands, within-patch compositional diversity arises if habitat patches contain different vegetation types or associations [31] and we thus interpreted compositional HD within patches as the diversity of plant associations within habitat patches (vegetation patchiness, Table 1).

**Table 1:** An Overview of the Terminology and the Definitions of Variables Used to Describe Habitat Diversity.

Scale	Factor	Variable	Description
Between patches	Compositional	Habitat type	Alkali marsh, wet meadow, alkali steppe, loess grassland
	Structural	Patch area	Area in hectares (log-scale)
		Patch shape	Patton's (1975) shape index
		Isolation	Distance to nearest similar patch (log-scale)
Within patch	Compositional	Vegetation patchiness	Exponential of Shannon-diversity of plant associations
	Structural	Vegetation height	Mean of min. 15 measurements per patch
		Bare ground cover	Cover of non-vegetated surfaces (%)

Structure is most often viewed as the configurational-architectural complexity of the abiotic and biotic elements that comprise the habitat [16]. We thus interpreted structural HD between patches as the variation in the architectural complexity (area, shape, isolation) of the habitat types. Although intensively managed grasslands are frequently envisioned as simple two-dimensional habitats, native or semi-natural grasslands are often multi-layered habitats holding various life-forms such as grasses, sedges, rushes, herbs, forbs, and shrubs [31]. In such grasslands, within-patch structural HD arises if the patches contain architecturally complex abiotic elements (slopes, rocks), vegetation patterns (vertical layers or horizontal mosaics) or various life-forms. Therefore, we interpreted within-patch structural HD as the variation in abiotic gaps (bare ground surfaces) and complexity of vegetation structure (measured by vegetation height) within the habitat patches (Table 1).

### Study site

We tested the effects of HD on SR of various taxa in a semi-natural grassland ecosystem of Hortobágy National Park, E-Hungary. Hortobágy, one of the largest steppes and most unique areas in Europe, lies in the NE part of the Carpathian Basin in Central/Eastern Europe. Our study area is in the Egyek-Pusztakócs marsh and grassland system, a 5000-ha isolated unit of Hortobágy National Park, the oldest (1973-) and largest (82 000 ha) national park in Hungary. The entire park is a World Heritage Site and is included in the Natura 2000 network of habitats in Europe as the classic locality of two priority habitat types of European importance (Pannonic loess steppic grasslands and Pannonic alkali grasslands and marshes) listed in Annex I of the Habitats Directive of the European Union [36].

**Fig 1:**
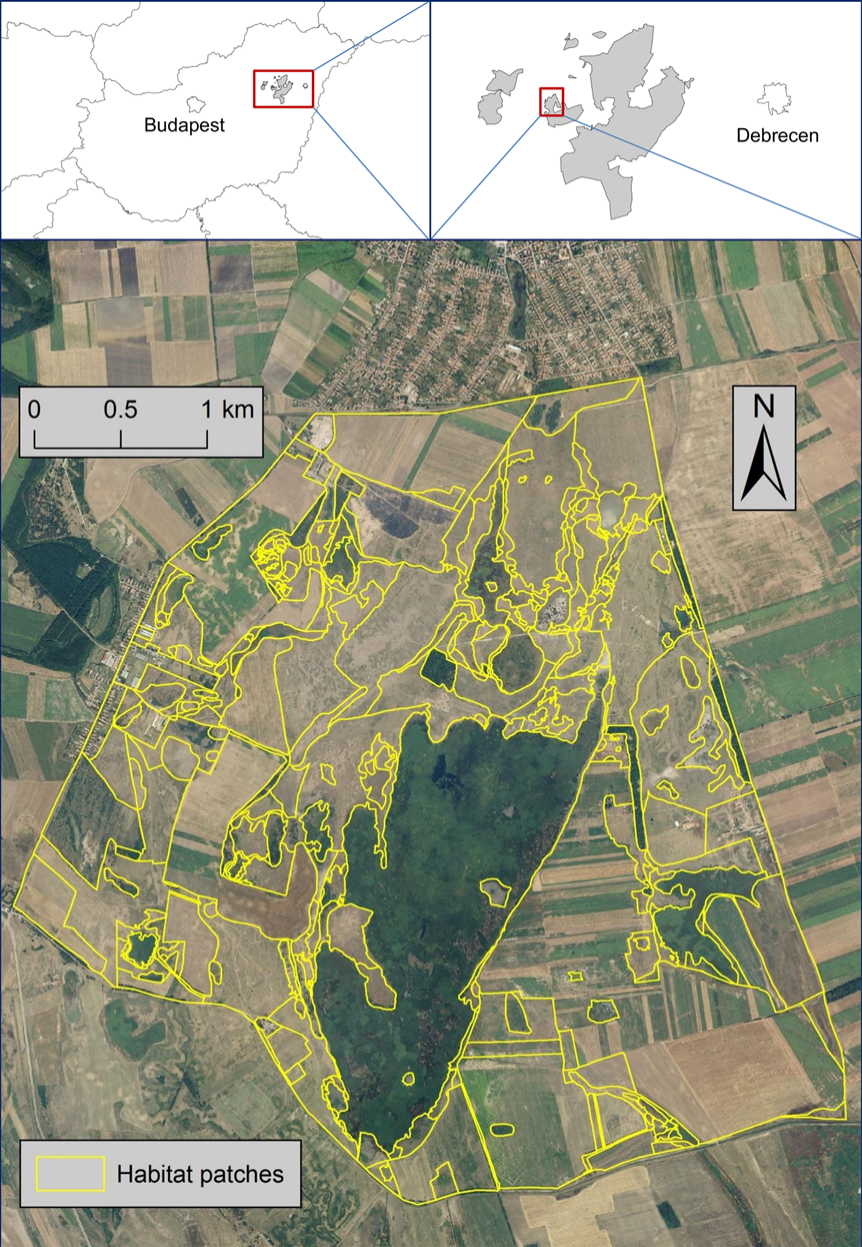
Location of Hortobágy National Park in Eastern Hungary (Left Insert), Location of the Study Area Within Hortobágy National Park (Right Insert), and Habitat Patches in the Study Area South of the Village of Egyek (47°37'32.9"N, 20°53'26.6"E).

The study area covers 1500 ha in the NW part of the Egyek-Pusztakócs habitat complex, and is a mosaic of alkali marshes, wet meadows, alkali steppes and loess grasslands (Fig 1).

All habitat types characteristic to the Hortobágy region are represented and concentrated in the relatively small and geomorphologically diverse study area. The differences among habitat patches arise largely due to small-scale differences in microtopography (relief), exposure to water table fluctuations (water balance), and soil quality, which lead to a formation of a mosaic pattern of different habitat types [37]. The study area has been managed by low-intensity farming (cattle-grazing or mowing) through renting state-owned lands to local farmers since 1973. The rental contracts between the national park directorate and the farmers specify various restrictions that ensure that conservation enjoys priority over other land uses [38].

### Field methods

We measured HD and sampled plants and animals in 51 habitat patches south of the township of Egyek (N 47°63', E 20°89') between May and September in 2004. The entire study site is within Hortobágy National Park, and fieldwork was commissioned, supported and funded by Hortobágy National Park Directorate (HNPD), the governing authority of the national park, through a LIFE-Nature project awarded by the European Commission to HNPD (LIFE04NAT/HU/000119, http://life04.hnp.hu) for which this study served as a baseline survey. Habitat patches were delineated from aerial photographs and were verified and classified in the field according to the General National Habitat Classification System (ÁNÉR) of Hungary [39] as (i) alkali marshes, (ii) wet meadows, (iii) alkali steppes and (iv) loess grasslands. The ÁNÉR classification is based on vegetation; therefore, our concept of habitat type also serves as a proxy for the physico-chemical factors that determine vegetation composition [32]. Marshes are covered by water (max. depth 0.5 m) most of the year, while wet meadows, which typically surround marshes, are covered by water from late winter to early summer. Alkali steppes are short-grass prairies on non-inundated alkali soils and loess grasslands are tall-grass prairies typically on richer chernozem soils of higher grounds, most of which have been converted to croplands. The vegetation of each of the four habitat types are potential keystone structures, i.e., spatially distinct units of vegetation that cause increases in species diversity by providing shelter to numerous insect species [9].

We selected habitat patches for this study based on a stratified random design. We randomly selected patches in three habitat types (alkali steppes, wet meadows, marshes) until we had a good representation of the variation in patch area, and added all four loess grassland remnants. The area of the patches ranged over two orders of magnitude (0.7 – 35.8 ha; mean: 9.1 ha; total area selected: 462 ha). There was no spatial autocorrelation in habitat types among the patches selected (Moran’s I index = -0.02, Z score = -0.202, p > 0.05).

We surveyed flowering plants in the selected habitat patches in late June, when the identification of such plants is most feasible. For the botanical survey, we randomly selected three 2×2 m plots per patch. To reliably sample plant species, we surveyed additional, also randomly selected, plots if the heterogeneity of the habitat patch was high. Botanical survey was conducted in a total of 231 plots (mean 4.5 ± SD 1.54 plots per patch, range: 3–9). The number of surveyed plots was not related to the area of the patch (Spearman rho = 0.210, p = 0.152), indicating that sampling effort and area were not confounded. We determined every plant to species within the plots using Simon [40] and estimated their cover; plant associations were identified based on Borhidi [41].

In the zoological survey, we sampled true bugs Heteroptera, grasshoppers-katydids Orthoptera, and vegetation-dwelling spiders Araneae (vegetation-dwelling arthropods); ground beetles Carabidae, ground-dwelling spiders Araneae (ground-dwelling arthropods); and birds Aves. Vegetation-dwelling arthropods were sampled by 100 strokes with a sweepnet in a 3×3 m plot enclosed by vertical, 1-m-high plastic foil installed to prevent arthropods from escaping. Plots were selected randomly in each of the 51 habitat patches. Ground-dwelling arthropods were sampled by Barber pitfall traps in two randomly selected points in 26 habitat patches. Traps were 0.5 L plastic cups filled with 10 mL ethylene-glycol as killing liquid, and were covered by fiberboard. To ensure the robustness of arthropod SR estimates to phenological/seasonal changes, we conducted sweep-netting and emptied pitfall traps once every three weeks from mid-June to late September (six occasions total). The insects collected by sweep-netting and pitfall trapping were sorted and identified to the species level in the laboratory.

Birds were censused at one counting point per patch in 51 habitat patches twice in the season (late April-early May and early June) according to the protocol of the Hungarian Common Bird Monitoring Scheme [42]. Counting points were designated in the centre of each habitat patch. During counting, all birds using the habitat patch for any activity (nesting, feeding/hunting, resting etc.) were counted in a circle of 100 m radius from the observation point for 5 min. Species recorded in either of the two counts were tallied to estimate bird SR.

### Variables and data analysis

Independent variables describing structural and compositional factors of HD at the patch and within-patch level are summarised in Table 1. The area of the habitat patches was measured in ArcGIS 9.0 and was log-transformed for statistical analysis. We characterised patch shape by Patton’s shape index, which provides an area-independent descriptor of shape by comparing patch shape to a circle of similar area [43]. Vegetation patchiness was expressed as the effective number of associations *e*^*HS*^, where *HS = -∑p*_*i*_*·logp*_*i*_, where *p*_*i*_ is the relative cover of the *i*-th association in the patch [44]. Vegetation height was measured at five points in each plot (accuracy 0.05 m) and averaged for plots, whereas bare ground cover was estimated in the field as the cover of non-vegetated surfaces.

We used general linear models (GLM) to test the effects of HD, area and management type on SR. Correlation analyses using all possible pairs of continuous independent variables revealed no evidence of multicollinearity (Pearson correlations, p > 0.05). Also, within-patch variables were generally not correlated with between-patch variables, with two exceptions. First, vegetation height was lower in dry than in wet habitat types (alkali steppes: 25.5 ± 14.55 cm, n = 23; loess grasslands: 33.8 ± 9.74, n = 4; wet meadows: 46.7 ± 20.86 cm, n = 12; alkali marshes: 117.8 ± 30.35 cm, n = 12; one-way ANOVA on log-transformed data, F_3,47_ = 19.748, p < 0.001). Second, dry habitat patches were larger than wet patches (loess grasslands: 15.2 ± 12.96 ha, n = 4; alkali steppes: 13.4 ± 11.43 ha, n = 23; marshes: 4.7 ± 5.06 ha, n = 12; wet meadows: 3.0 ± 1.44 ha, n = 12; one-way ANOVA on log-transformed data, F_3,47_ = 8.735, p < 0.001). In cases when either of the related variables were significant in the full model or the final reduced model (see below), we refitted the final GLMs with either or both of the related variables to evaluate their relative effect on SR. Vegetation patchiness did not differ among habitat types (one-way ANOVA, F_3,48_ = 2.562, p > 0.05), indicating that habitat types were similarly heterogeneous regarding their plant species composition. Finally, although most patches (n = 30) were not managed, some patches were managed by low-intensity cattle-grazing (n = 17) or mowing by machine in late June (n = 4). To control for potential variation due to different management practices, we included the effect of management in statistical analyses as a categorical variable with three levels (no management, grazing, mowing).

In all analyses, response variables were SR estimates of major combined groups (all species, all animal species, plants, vegetation-dwelling arthropods, ground-dwelling arthropods) or taxonomic groups separately. Data from sampling occasions (n = 6 for arthropods, n = 2 for birds) were pooled to account for phenological changes in SR. To account for potential biases induced by differences in detection probability of birds [45], we obtained estimates of richness that account for heterogeneous detection probabilities using a jackknife estimator [46] as implemeted in the COMDYN software (http://www.mbr-pwrc.usgs.gov/software/comdyn.html). The results obtained using the corrected estimates provided results qualitatively similar to those presented here and did not change our conclusions. Because sampling effort was adjusted for patch heterogeneity for plants but not for animals, we also performed all analyses using data from a fixed number of botany plots only (n = 3 randomly selected plots per patch). Although plant species numbers decreased slightly (5–10%), the resulting models were qualitatively similar to those presented here and we thus concluded that our results were not affected by the adjusted sampling effort.

Model selection was started by fitting the full GLM containing all independent variables and their biologically relevant interactions. In GLMs testing total and plant SR, independent variables were patch area, patch shape, isolation and management type. In GLMs testing all other variables (animal SR in major groups and single taxa), independent variables were habitat type, patch area, patch shape, isolation and management type (between-patch level) and vegetation height, bare ground cover and vegetation patchiness (within-patch level, Table 1). We then implemented a backward stepwise variable removal algorithm (p_out_ > 0.05) to obtain a final reduced minimum adequate model. We used the final reduced models to obtain parameter coefficients for continuous independent variables and to carry out post-hoc comparisons using Tukey’s HSD procedure for categorical independent variables. Next we also fitted the models containing either between-patch or within patch-level independent variables only and used the same backward stepwise algorithm to test model fit at the two different scales separately. We have also tested the biologically meaningful interactions in all final models. None of the interaction terms were significant (p > 0.1), therefore, we present results with main effects only. Finally, we tested whether spatial autocorrelation affected observations of the response variables by calculating Moran’s I values based on a spatially weighted neighbour matrix of each response variable, as suggested by Dormann et al. [47]. Residuals of neither response variable showed significant spatial autocorrelation (p > 0.05), therefore, we did not control for such autocorrelation in GLMs. The normality of residuals was checked by normal probability plots and Kolmogorov-Smirnov tests and the homogeneity of variances was tested using Levene’s test. When these assumptions were not met, we log-transformed variables before analysis. In GLMs, Type II sums of squares (SS) were specified to prevent biases due to entry-order sensitivity present in models using Type I and Type III SS and because our primary aim was model selection, for which Type II SS is more appropriate [48]. We used two-sided tests and α = 0.05 significance levels in statistical tests. Means ± S.D.s are given in the text, except where indicated. All statistical analyses were performed using the R statistical language and environment, version 3.2.2 [49].

## Results

### Total species richness and habitat diversity

A total of 434 species were recorded in the 51 habitat patches (Table 2). Most species were arthropods (221 species) or flowering plants (170 species). The average number of species per patch was roughly similar in the three major groups (17–22 species per group per patch). Total SR (all groups combined) was not related to any measure of HD or area as the final model contained only the intercept term (Table 3).

**Table 2:** Number of Species by Major Groups and Taxa and Number of Species per Habitat Patch in the Egyek-Pusztakócs Marsh-Grassland System in E Hungary. N indicates number of habitat patches surveyed.

Major group	Taxon	Number of species			N
		Total	Mean ± S.D.	Min—Max	
Plants	Flowering plants	170	21.3 ± 11.64	2 - 46	51
Vegetation-dwelling arthropods	Araneae	65	8.3 ± 4.07	1 - 21	51
	Heteroptera	14	1.7 ± 1.17	0 - 5	51
	Orthoptera	32	7.7 ± 2.58	2 - 13	51
	Subtotal	111	17.7 ± 5.52	7 - 31	51
Ground-dwelling arthropods	Araneae	43	9.2 ± 6.98	0 - 25	26
	Carabidae	67	12.8 ± 8.56	2 - 33	26
	Subtotal	110	22.0 ± 14.18	2 - 49	26
Birds	Aves	43	3.7 ± 2.50	1 - 13	51
Total species richness		434	67.0 ± 15.10	39 - 98	26

**Table 3:** Minimum Adequate General Linear Models Testing the Relationship Between Different Factors of Habitat Diversity and Species Richness of Major Groups. Significant (p < 0.05) effects and significance levels are shown in bold.

Major group	Scale	AIC ^a^	Variables in final model	F	p	Relationship/difference ^b^
All taxa sampled	Between-patch	151.39	None (intercept only)	–	–	none
All animals (log)	Both scales	-71.41*	**Vegetation height**	8.280	**0.009**	positive correlation
			Isolation	3.061	0.094	
			Bare ground cover	2.768	0.110	
	Between-patch	-66.80*	**Habitat type**	3.305	**0.040**	marshes > alkali steppes
			Isolation	1.883	0.184	
	Within-patch	-70.08*	**Vegetation height**	11.528	**0.002**	positive correlation
Vegetation-dwelling arthropods	Both scales	171.19*	**Habitat type**	2.858	**0.048**	loess grassland > meadow
			Isolation	2.598	0.114	
			**Management type**	**3.308**	**0.046**	(non-managed > grazed)
			Vegetation patchiness	3.919	0.054	
	Between-patch	175.24	**Habitat type**	3.143	**0.035**	loess grassland > meadow
			Isolation	1.764	0.191	
			Management type	3.042	0.058	
	Within-patch	177.13	**Vegetation patchiness**	4.102	**0.048**	positive correlation
Ground-dwelling arthropods (log)	Both scales	-22.60*	**Habitat type**	4.359	**0.016**	marshes > alkali steppes
			**Bare ground cover**	**4.729**	**0.042**	positive correlation
			Isolation	3.757	0.067	
	Between-patch	-20.53*	**Habitat type**	4.734	**0.011**	marshes > alkali steppes
	Within-patch	-17.83	**Vegetation height** Bare ground cover	6.5152.039	**0.018**0.167	positive correlation

^a^ Akaike’s Information Criterion and model significance

* p < 0.05

** p < 0.01

*** p < 0.001

^b^ Tukey test for categorical variables (p < 0.05; parentheses indicate marginally non-significant difference).

### Scale-dependent correlations between species richness and habitat diversity

Models containing both between-patch and within-patch variables had the lowest AIC values for each response variable (Tables 3 and 4). For orthopterans, true bugs and ground beetles, the model containing variables at both levels and the model containing only between-patch variables resulted in the same final model (Table 4), indicating that SR of these taxa was primarily related to larger-scale variables. In contrast, the final models for spiders and birds also included one within-patch variable (vegetation-dwelling spiders, birds: vegetation patchiness; ground-dwelling spiders: vegetation height) or more within-patch variables (ground-dwelling spiders: vegetation height, bare ground cover) (Table 4), suggesting the importance of finer-scale HD in the richness of these taxa.

**Table 4:** Minimum Adequate General Linear Models Testing the Relationship Between Different Factors of Habitat Diversity and Species Richness of the Studied Taxa. Significant (p < 0.05) effects and significance levels are shown in bold.

Taxon	Scale	AIC ^a^	Variables in final model	F	p	Relationship/difference ^b^
Flowering plants	Between-patch	243.92**	Patch area	1.899	0.175	
			**Management type**	3.291	**0.046**	(grazed > non-managed)
Vegetation-dwelling spiders	Both scales	137.16**	**Vegetation patchiness**	8.419	**0.006**	positive correlation
			Management type	2.372	0.104	
	Between-patch	141.80	Habitat type	2.758	0.053	(marsh > meadow)
	Within-patch	138.07**	**Vegetation patchiness**	8.316	**0.006**	positive correlation
Orthopterans	Both scales	89.00**	**Habitat type**	5.179	**0.004**	loess grassland > meadow = marsh
	Between-patch	89.00**	**Habitat type**	5.179	**0.004**	loess grassland > meadow = marsh
	Within-patch	97.44*	Bare ground cover	2.084	0.155	
True bugs (log)	Both scales	-83.71**	Patch shape	3.591	0.064	(negative correlation)
			**Isolation**	10.210	**0.003**	negative correlation
			**Management type**	5.623	**0.007**	non-managed > grazed
	Between-patch	-83.71**	Patch shape	3.591	0.064	(negative correlation)
			**Isolation**	10.210	**0.003**	negative correlation
			**Management type**	5.623	**0.007**	non-managed > grazed
	Within-patch	-74.28	Bare ground cover	0.574	0.452	none
Ground beetles	Both scales	97.89***	**Habitat type**	9.084	**0.000**	marshes = meadows > alkali steppes
			Vegetation patchiness	1.859	0.188	
			Bare ground cover	1.626	0.217	
	Between-patch	98.70**	**Habitat type**	8.444	**0.001**	marshes = meadows > alkali steppes
	Within-patch	101.63***	**Vegetation height**	15.563	**0.001**	positive correlation
Ground-dwelling spiders (log)	Both scales	-5.51*	Habitat type	2.128	0.134	
			Isolation	3.341	0.085	(positive correlation)
			Management type	2.133	0.149	
			Vegetation height	2.873	0.108	
			**Bare ground cover**	7.835	**0.012**	positive correlation
	Between-patch	99.84	Isolation	4.221	0.051	(positive correlation)
	Within-patch	100.66	Vegetation height	3.339	0.080	(positive correlation)
Birds (log)	Both scales	-50.23**	**Patch area**	12.172	**0.001**	positive correlation
			**Vegetation height**	5.548	**0.023**	positive correlation
			Vegetation patchiness	3.754	0.059	(positive correlation)
	Between-patch	-45.97	**Patch area**	6.966	**0.011**	positive correlation
			Habitat type	2.324	0.087	(marsh > meadow)
	Within-patch	-41.10	Vegetation patchiness	2.194	0.145	

^a^ Akaike’s Information Criterion and model significance

* p < 0.05

** p < 0.01

*** p < 0.001

^b^ Tukey test for categorical variables, marginally significant differences are shown in parentheses.

**Fig 2:**
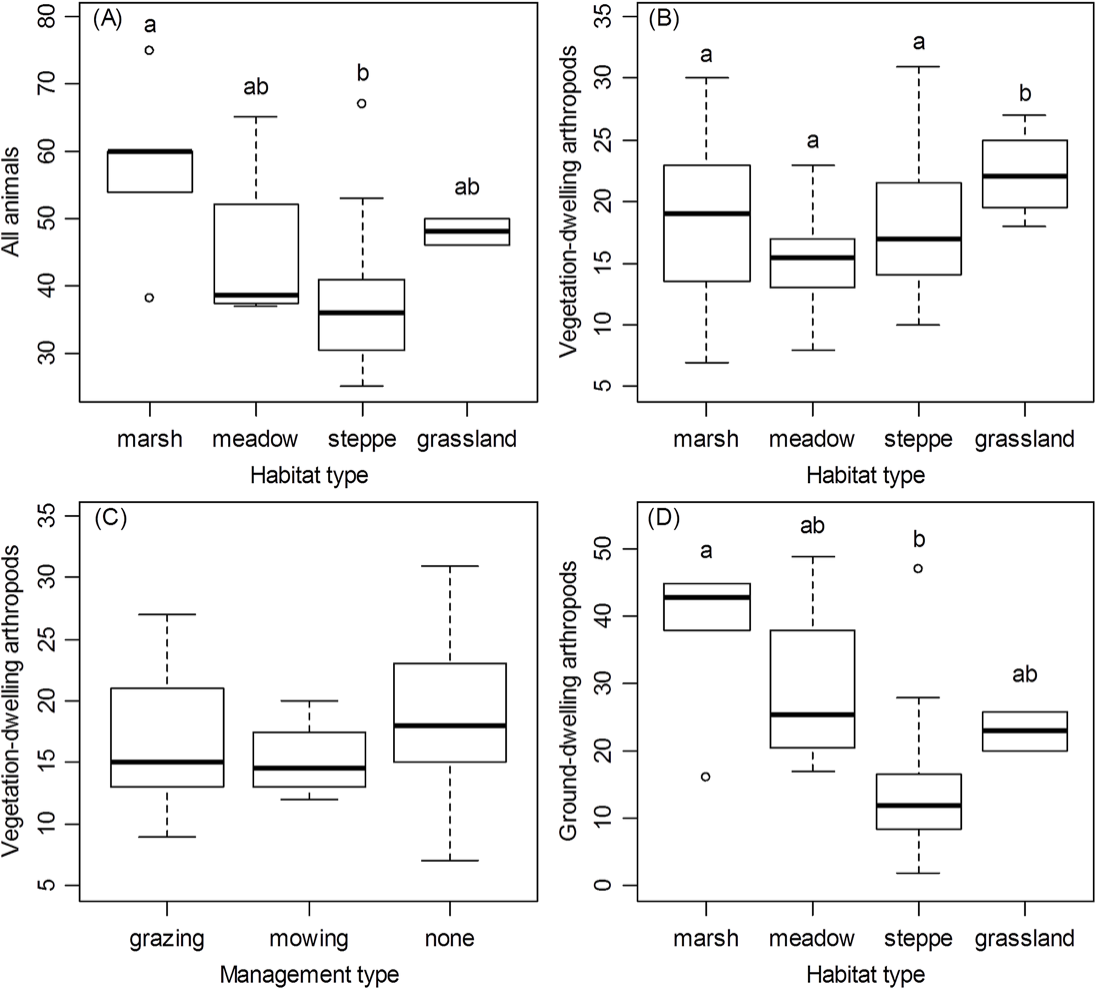
**Species Richness of All Animals (A), Vegetation-dwelling Arthropods (B, C), and Ground-dwelling Arthropods (D) in Various Types of Habitat (A, B, D) and Management (C).** Boxplots show the median (horizontal line), the 25th and 75th percentiles (bottom and top of box, respectively), the minimum and maximum values (whiskers, in case of no outliers) or 1.5 times the interquartile range (whiskers, in case of presence of outliers) and outliers (dots). Groups not sharing lowercase letters are significantly different (Tukey’s HSD test, p < 0.05; additional statistics are given in Table 3).

**Fig 3:**
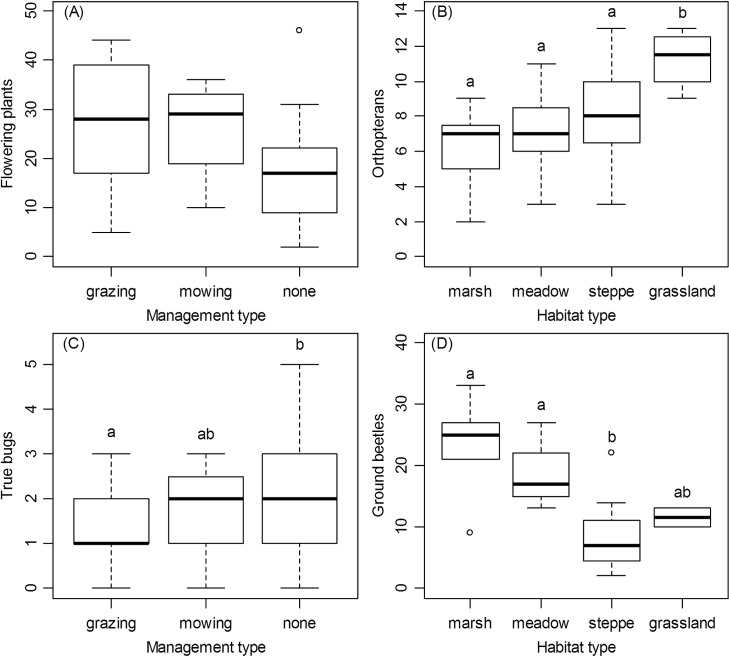
**Species Richness of Flowering Plants (A), Orthopterans (B), True Bugs (C) and Ground Beetles (D) in Various Types of Management (A, C) and Habitat (B, D).** Groups not sharing lowercase letters are significantly different (Tukey’s HSD test, p < 0.05; additional statistics are given in Table 4).

At the between-patch scale, the wet-dry habitat gradient, i.e., habitat type, showed the strongest correlations with SR (all animals, vegetation-dwelling and ground-dwelling arthropods, orthopterans, and ground beetles) (Tables 3 and 4). Marshes, the wettest habitat type, had significantly more animal species than the considerably drier alkali steppes, whereas meadows and loess grasslands had intermediate richness (Fig 2**A**). There were more vegetation-dwelling species in dry loess grasslands than in wetter habitats (Fig 2**B**), mostly due to higher SR of orthopterans in loess grasslands than in wet habitats (Fig 3**B**). The relationship was opposite for ground-dwelling arthropods (Fig 2**D**), mostly because of higher SR of ground beetles in wet habitats (marshes and meadows) (Fig 3**D**). Management was related to SR of flowering plants, vegetation-dwelling arthropods, and true bugs in particular (Tables 3 and 4, Figs 2 and 3). Flowering plants were influenced by management and had more species in grazed patches than in non-managed patches (Fig 3**A**), although the difference was marginally non-significant (Tukey test, p < 0.1). In contrast, true bugs had more species in non-managed than in grazed patches (Fig 3**C**). Finally, patch area was positively related to bird SR, and isolation was negatively related to true bug SR, indicating fewer species in more isolated patches (Table 4). None of the other variables at the between-patch scale (patch area, shape and isolation) were significant (Table 4).

**Fig 4:**
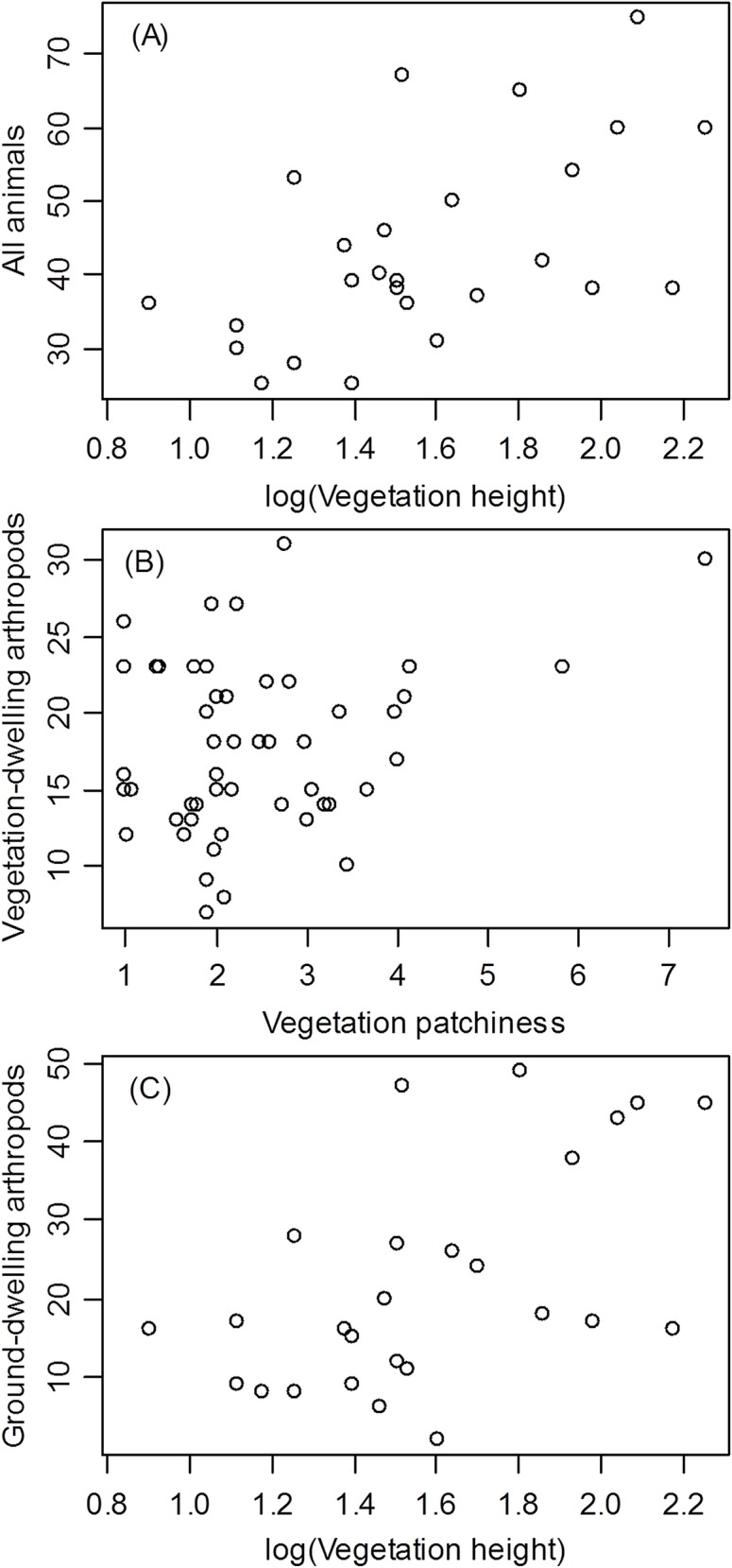
**Species Richness of All Animals (A), Vegetation-dwelling Arthropods (B), and Ground-dwelling Arthropods (C) as a Function of Vegetation Height (A, C) or Vegetation Patchiness (B).** GLM, (A): B = 0.48 ± (S.E.) 0.141, F_1,24_ = 11.528, p = 0.002; (B): B = 1.25 ± 0.619, F_1,49_ = 4.102, p = 0.048; (C): B = 0.86 ± 0.387, F_1,23_ = 6.515, p = 0.018).

**Fig 5:**
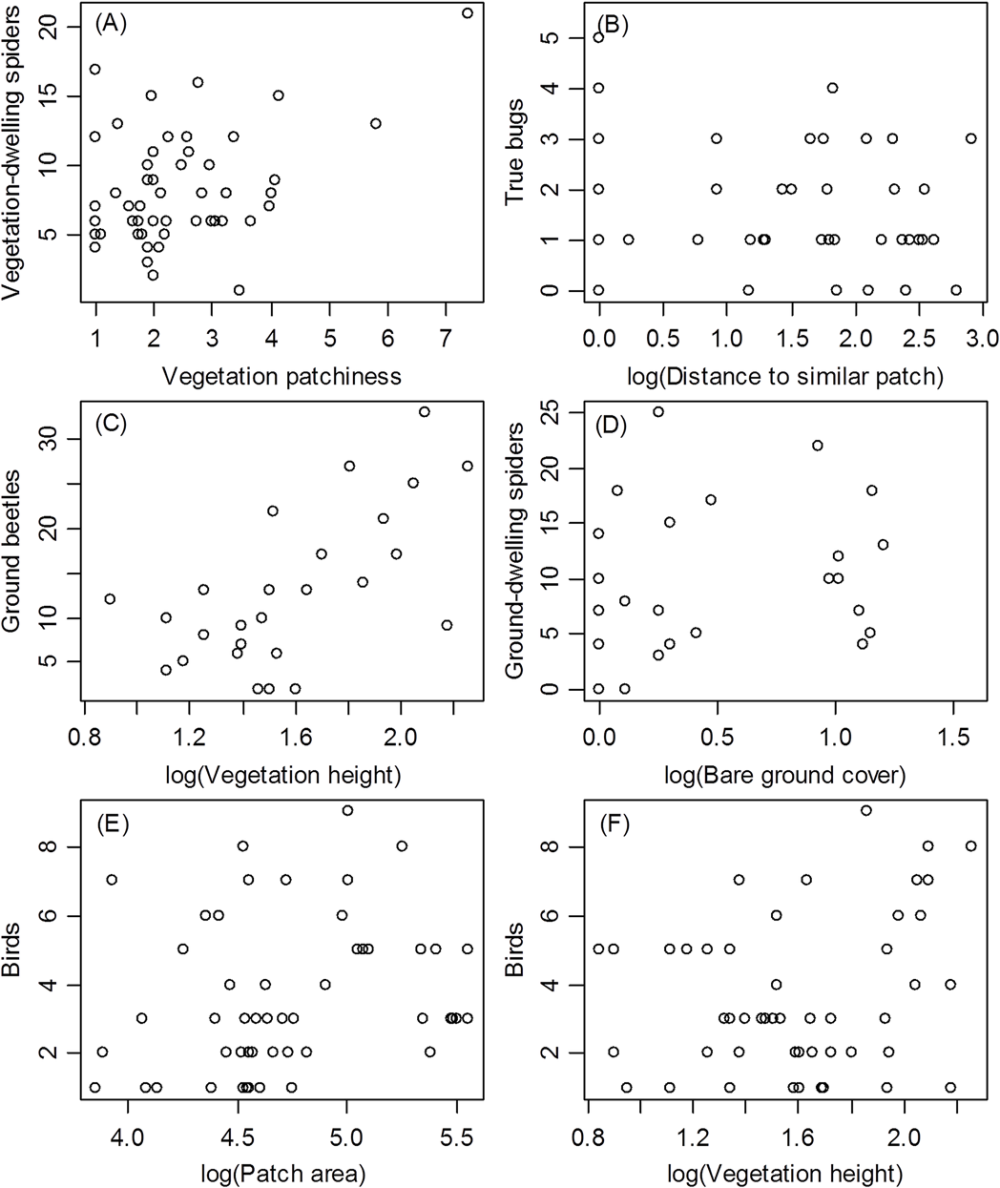
**Species Richness of Vegetation-dwelling Arthropods (A), Ground Beetles (B), Birds (C, F), True Bugs (D), and Ground-dwelling Spiders (E) as a Function of Habitat Diversity Variables Found to be Significant in General Linear Models (Table 4).** GLM, (A): 1.26 ± 0.438, F_1,49_ = 8.316, p = 0.006; (B): B = -0.21 ± 0.066, F_1,46_ = 10.210, p = 0.003; (C): B = 15.14 ± 3.837, F_1,24_ = 15.563, p < 0.001; (D): B = 1.05 ± 0.374, F_1,17_ = 7.835, p = 0.012; (E): B = 0.63 ± 0.238, F_1,46_ = 6.966, p = 0.011; (F): B = 0.59 ± 0.249, F_1,47_ = 5.548, p = 0.023.

At the within-patch scale, vegetation height had the strongest relationships with SR (all animals, ground-dwelling arthropods, ground beetles, birds) (Tables 3 and 4, Figs 4 and 5). Vegetation patchiness was also related to SR in the case of vegetation-dwelling arthropods, and vegetation-dwelling spiders in particular, the richness of which increased with vegetation patchiness more than with any other measure of HD (Tables 3 and 4, Figs 4 and 5).

All correlations between combined or taxon SR and HD variables were positive (Tables 3 and 4, Figs 4 and 5), indicating that SR generally increased with HD.

## Discussion

Our study provided three key results. First, we found positive correlations between HD and SR in each taxon and major group studied, supporting the view that SR generally increases with HD. Second, taxa and major groups differed in what measure of HD their SR showed positive correlations with. As predicted, compositional HD (vegetation patchiness) mostly affected the richness of vegetation-dwelling, mostly herbivorous, arthropods, whereas structural HD (vegetation height) was more related to SR of ground-dwelling, mostly predatory arthropods, and birds (Tables 3 and 4). Finally, as a consequence of the idiosyncratic relationships between taxon or group SR and HD, we found no clear relationship between total SR (all groups combined) and HD. In summary, our findings provide empirical evidence that even though HD positively influences SR in a wide range of grassland taxa, each taxon responds to different compositional or structural measures of HD, resulting in the lack of a consistent relationship between HD and SR when taxon responses are pooled.

### Compositional/structural factors of habitat diversity and single taxa

Our study is one of the first attempts to integrate compositional and structural factors of HD at the between-patch and within-patch scales and to demonstrate responses of plant and animal SR to these different measures in grasslands. Although we find that the major groups and taxa respond to different measures of HD, the taxon-specific responses found here are not unique in our grassland system and generally agree with those reported previously in other studies. For example, HD was the most important predictor of SR of various arthropod groups in nature reserves in south-central Hungary [20]. Other studies of semi-natural grasslands also found that floristic composition was a better predictor of arthropod richness than vegetation structure or environmental conditions [32, 50]. The positive correlation found between vegetation patchiness and SR of vegetation-dwelling spiders is compatible with previous observations on spiders in forests, where SR was primarily influenced by structural HD [16, 51–53]. Our findings on ground beetles are similar to those of Brose [34], who found that vegetation structure, i.e., more prey, shade or humidity in complex micro-environments, is more important for predatory ground beetles than the taxonomic diversity of plants. Structural HD was also found previously to influence SR of orthopterans [54] and true bugs [55], which we also found in our study. Birds were also more species-rich in more complex habitat patches with taller vegetation, which likely have more microsites available for feeding and nesting than patches with shorter vegetation [56]. For some other taxa, however, our results contradicted those of previous studies. For example, structural diversity created by different management, rather than compositional diversity, is known to influence the diversity of many vegetation-dwelling taxa, such as true bugs [57]. In our study, grazing was associated with lower SR in true bugs and higher richness in plants but it did not significantly affect other groups or taxa (Table 3).

### Species-area vs. species-habitat diversity relationships

Patch area was positively related to SR only for birds and not for any other taxon or major group. The positive SAR for birds may be explained by heterogeneity within the patch or by heterogeneity of the surrounding environment. The first is unlikely because no within-patch HD factor was significant in the reduced model for birds (Table 4), whereas the second is likely if larger patches have more neighbours than smaller patches. The lack of a significant SAR in other groups suggests that the scale at which our study was conducted (1–36 ha) was below the scale at which SARs become important in explaining diversity patterns. The exact scale at which HD becomes influential over the SAR is rarely studied empirically [2], although the prevalence of the small island effect (no increase in species number with area at small scales) in many real archipelagoes [58] suggests that such a cut-off point does exist. Our study shows that HD can be at least as important as area effects in explaining patch-level patterns in biological diversity at local scales. Consequently, this study provides an example when a SAR detected in one group may not be present in other taxa, which are primarily influenced by other factors, beyond the simple effect of area [20, 59–61]. Alternatively, it is also possible that the relative importance of HD varies by spatial scale across taxa. For instance, organisms perceive their environment at different spatial scales and the grain size of the environment will be different for species in different taxa or with different body size [9, 62]. This explanation suggests that the scale of our study was large enough to detect areal effects in some groups (i.e., birds) and was small enough to detect HD effects in other groups. The results thus call for the need to incorporate both HD and the SAR in studies attempting to explain overall biological diversity at the patch-level. One practical difficulty with such an integration is that there is no readily available metric to measure HD [9] and that the influence of different factors on biodiversity may vary across spatial scales [63, 64]. As this study attests, even though simple measures of HD can be suitable to detect relationships in certain taxa, it is less straightforward how to unite these simple measures into a general index of HD. Our framework may contribute to the development of such a metric by providing an example for separating and explicitly addressing the structural and the compositional factors of HD at two spatial scales.

### Keystone structures and management implications

Our findings provide further insight into the relevance of keystone structures, or distinct spatial structures that provide resources such as food and shelter to groups of other species (e.g. deadwood in forests [9]), in grassland ecosystems. The richness of ground-dwelling arthropods was significantly higher in wet habitats (marshes, meadows) than in dry ones (steppes, grasslands), whereas an opposite relationship was found for vegetation-dwelling arthropod species. Thus, our results suggest that lower-lying wetter areas appear as keystone structures for ground-dwelling, mainly predatory, groups and that dry grasslands may function as keystone structures for vegetation-dwelling, mainly herbivorous, groups. This study thus shows that different keystone structures may exist in the same landscapes for different taxonomic or trophic groups. These keystone structures need to be identified and preserved or managed appropriately for the conservation of landscape-level biodiversity [9].

Our results draw attention to the risks of decreasing HD for conservation: the decrease in HD, or the homogenization of habitats, can lead to a decrease in several components of biological diversity. This threat is currently less well recognised than the direct threats arising from the area loss of habitats or from habitat fragmentation. Relationships found in this study suggest that although different taxa respond to different factors of HD, SR increases with HD in all taxa [9, 57, 65]. Therefore, the results also suggest that the creation or restoration of different habitat types within a landscape may lead to an increase in patch-level species diversity. For example, management of homogeneous reedbeds by burning and cattle-grazing may effectively increase HD in marshes and can have beneficial effects both for amphibians and birds [66, 67]. Our results, therefore, support the view that the best way to preserve or enhance biological diversity is to maintain a complex, mosaic-like landscape structure which has ideal conditions for most of the organisms [30, 56, 68, 69]. Conservation actions, therefore, should aim to increase both patch-level HD, e.g. by increasing the number of different habitat types through creation or restoration, and within-patch level diversity, e.g. by enhancing within-patch mosaic pattern through adaptive ecosystem management [66, 67]. The maintenance of such artificial heterogeneity may be advisable in evidence-based conservation when the establishment and persistence of species or habitat types of conservation concern is associated with heterogeneity.

### Conclusions

In conclusion, our results show that species diversity is positively related to HD at small scales (between and within habitat patches in a landscape). However, there is no general relationship because different factors of HD influence different components of biodiversity. The wet-dry habitat types explained most variation in arthropod SR at the between-patch level. Compositional HD mostly influenced vegetation-dwelling spiders, whereas structural HD mostly affected ground-dwelling taxa and birds. HD, therefore, should be incorporated along with area effects in studies of small- to mid-scale diversity patterns and HD should be the focus of habitat management to maintain landscape-scale biodiversity in grassland ecosystems.

## Supporting Information

S1 Table Dataset that provided the basis for the analyses presented. (XLS)
